# Cows Come Down from the Mountains before the (M_w_ = 6.1) Earthquake Colfiorito in September 1997; A Single Case Study

**DOI:** 10.3390/ani4020292

**Published:** 2014-06-03

**Authors:** Cristiano Fidani, Friedemann Freund, Rachel Grant

**Affiliations:** 1 Osservatorio Sismico “Andrea Bina”, Borgo XX Giugno 74, 06121 Perugia, Italy; 2 Central Italy Electromagnetic Network (CIEN), Via Fosso del Passo 6, 63847 San Procolo, Fermo, Italy; 3 Ames Research Center, National Aeronautics and Space Administration (NASA), Earth Science Division, Code SGE, Moffett Field, CA 94035, USA; E-Mail: friedemann.t.freund@nasa.gov; 4 Department of Physics, San Jose State University, San Jose, CA 95192, USA; 5 Carl Sagan Center, SETI Institute, 189 Bernardo Ave., Mountain View, CA 94043, USA; 6 Department of Life Sciences, Anglia Ruskin University, East Rd., Cambridge, CB1 1PT, UK; E-Mail: Rachel.grant@anglia.ac.uk

**Keywords:** animal behaviour, earthquakes, positive holes, air ionization, cows

## Abstract

**Simple Summary:**

Recent reports from several countries such as China, Italy and Japan support the existence of strange animal behaviour before strong earthquakes. However, the stimuli to which animals are sensitive preceding seismic activity are still not completely understood. Here we report the case of a herd of cows (reported by an entire village) leaving the hill pasture and descending near to the village streets two days before a strong earthquake.

**Abstract:**

The September–October 1997 seismic sequence in the Umbria–Marche regions of Central Italy has been one of the stronger seismic events to occur in Italy over the last thirty years, with a maximum magnitude of M_w_ = 6.1. Over the last three years, a collection of evidence was carried out regarding non-seismic phenomena, by interviewing local residents using a questionnaire. One particular observation of anomalous animal behaviour, confirmed by many witnesses, concerned a herd of cows, which descended from a mountain close to the streets of a village near the epicentre, a few days before the main shock. Testimonies were collected using a specific questionnaire including data on earthquake lights, spring variations, human diseases, and irregular animal behaviour. The questionnaire was compiled after the L’Aquila earthquake in 2009, and was based upon past historical earthquake observations. A possible explanation for the cows’ behavior—local air ionization caused by stress-activated positive holes—is discussed.

## 1. Introduction

Unusual animal behaviour prior to earthquakes has been observed for millennia [1], but until recently, there appeared to be no plausible explanation. Freund [2] presented a unified theory of non-seismic earthquake precursors, proposed on the basis of stress-activated positive hole charge carriers. It is likely that at least some of the observations of unusual pre-earthquake animal behaviour can be traced back to this underlying geophysical process. Other possible causes are the release of gases, such as CO, from the future epicentre [3], charged aerosol production [4], sound and vibrations [5], electric [6] and magnetic [7] field influences, ultra-low and extra-low-frequency electromagnetic effects [8,9]. It should be remembered that species-typical behaviours can be triggered by a variety of stimuli not necessarily related to earthquakes [10] and that sometimes animal behaviour anomalies reported by the public may actually not be abnormal behaviour [11]. Often much of the behaviour resembles that reported before other geophysical events, such as thunderstorms [12], or volcanic eruptions [13]. Animal behavior is very variable, even within the same species [14]. Finally, as there are geophysical differences between earthquakes [15], unusual animal behaviour is observed before some earthquakes but not others.

With this in mind, in this communication we present several incidences of unusual behaviour of cattle and also describe a previously unpublished observation of unusual cow behaviour prior to the Umbria-Marche earthquake (M_w_ = 6.1) of September 26, 1997. An evaluation in terms of the theory of positive holes leading to air ionization as proposed by Freund *et al.* [16] was applied to the Colfiorito tectonic structure in Central Italy, where the strong earthquake struck. 

**Figure 1:**
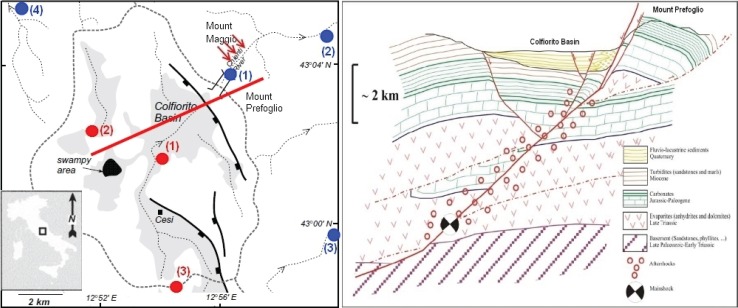
Colfiorito Basin. The red arrows in the left-hand box indicate cow movements, while red circles indicate the stronger shocks and blue circles indicate meteorological stations at Serravalle (1), Gelagna Alta (2), Pié del Sasso (3) and Bagnara (4) (the Nocera Umbra station is outside of the map to the west near Bagnara). A section of the Basin in the left-hand box is shown in red, it is represented in the right-hand box with the positions of the main shock and aftershocks along the normal fault plane in red; Mount Maggio is behind Mount Prefoglio, after Barchi [24].

The Colfiorito area is a closed extensional basin system located on the Apennines watershed and surrounded by valleys that are deeply incised into the Mesozoic–Cenozoic bedrock (Figure 1). The basin floor is at an elevation of 800 m whereas the level of river incision in nearby valleys rapidly decreases to 400 m within a few kilometres of the basin. Lacustrine and alluvial deposits containing remains of Lower Pleistocene mammal fauna [17] are exposed for 100 m in thickness in the central and southern parts of the basin. Surface geological data and seismic reflection profiles have clearly pointed out that the area of Colfiorito is structurally dominated by N–S and NNW–SSE trending macro-anticlines. Colfiorito plain is bounded to the NE by an active normal fault system [18] that ruptured during the 1997 Umbria-Marche earthquake sequence [19]. Despite the different interpretations given to the surface ruptures observed after the main shocks, the fault geometry revealed by seismological, geodetic, and field data [20,21] is consistent with the longer-term Quaternary evolution of the area. In particular, radar interferometry and GPS data showed several tens of centimetres of seismic subsidence in the hangingwall of the fault system bounding the basin [22], and surface ruptures of a few cm in amplitude were found after the earthquake along previously-mapped fault scarps at the foot of mountain fronts bounding the Quaternary basin [23].

The seismic sequence that affected the Umbria–Marche region (Central Italy) in the period September 1997–April 1998 caused the loss of 12 lives and severe damage to ca. 300 localities. Three main events took place at less than 10 km depth: two on September 26, 1997, (1) M_w_ = 5.8 and (2) M_w_ = 6.1, and one on October 14, 2007, (3) M_w_ = 5.6; indicated by red circles in Figure 1.

## 2. Methods

The L’Aquila earthquake has shown that the public is able to observe a large number of phenomena for which there were no available instruments at the time of the quake, including unusual animal behaviour [25]. The people of L’Aquila clearly remembered their observations for many months and in many cases they remembered observations from previous earthquakes. Hence, a collection of data was carried out relative to a strong earthquake which occurred some time before L’Aquila, that of Colfiorito.

Testimonies of unusual behaviour of wild and domestic animals were collected around Colfiorito Plain in connection with the September 1997 earthquake. The observations were collected in the months of September–October 2010, August–September 2011, May-August and October–November 2012, January and April 2013. People reported strange animal behaviour which had occurred in September 1997, *i.e.*, more than 13 years ago. It is possible that people’s memories are inaccurate and data may be unreliable after such as long period of time. However, it is also remarkable that so many people independently remembered and commented on the particular observation presented here. A questionnaire was used to obtain information on sightings of earthquake lights, with a small section on animal behaviour anomalies. The questionnaire was initially used to collect information relative to the L’Aquila earthquake in 2009 [26] (the full text can be downloaded from supplementary material [26]). Here, the section concerning animals is shown:
Did you observe strange behaviour on the part of animals? If yes, please describe the places, times and conditions:
55)Sudden death of domestic or stray animals?56)Yelping of dogs?57)Neighing of horses?58)Unexplainable appearance of ants?59)Abundant appearance of worms?60)Unexplainable appearance of snakes or frogs?61)Unexplainable appearance of mice?62)Unexplainable behaviour of chickens?63)Unexplainable behaviour of fish?64)Nervous behaviour of farming livestock?65)Sighting of rare animals?66)Disappearance of birds?67)Other strange animal behaviour? Please describe:
 Other, please describe:



This same questionnaire was used with the population of the regions of Umbria and Marche, affected by the strong earthquake of Colfiorito in September 1997. Questionnaires were not distributed on paper to the population but carried out by face to-face interviews, allowing as much information as possible to be collected [27]. A total of 94 people were interviewed in the Umbria-Marche region.

## 3. Observations and Results

### 3.1. Cows

**Figure 2:**
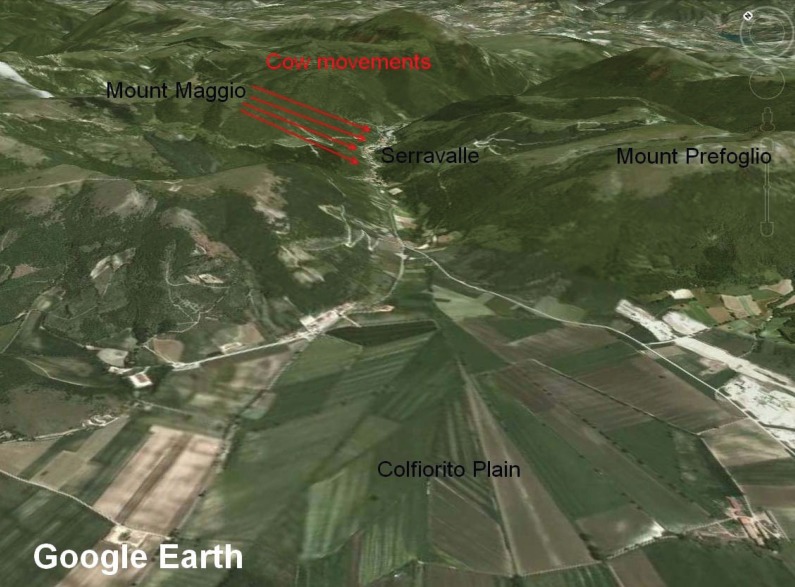
A Google Earth map of the Colfiorito Basin, red arrow indicates cow movements.

The observation on which we report here, was made in Serravalle del Chienti, a village located a few kilometers north-east of the earthquake epicentre of September 26, 1997, M_w_ = 6.1. Nineteen out of 94 people were interviewed in Serravalle, and of these, 17 reported behaviour which they considered anomalous, *i.e.*, cows descending to near the village before the earthquake. The cows were grazing on the top of Monte Maggio, located north of Serravalle, which is the village in a valley that separates it from Mount Prefoglio, see Figure 2. The cows are always let loose on the pastures (at Monte Maggio 1236 m) every summer. They return to their barn when the snow comes, usually at the end of November or beginning of December. The villagers know that the cows normally stay in the pasture area and do not leave it all summer. Two days before the main shock, however, the herd of cows was sighted near to the village of Serravalle del Chienti in the clearings above the highway 77 at 700 m. About 60 cows, the entire herd, had descended from the mountain (Figure 2) and into the town itself, which is a highly unusual behaviour, although all cows seemed to be in good health. The cows were seen by all the inhabitants of the village a few days before the earthquake. The reports confirmed that cattle descended from the mountain and stood in the area above the highway 77 for 4–5 days, after which time, the cattle went back up the mountain. The interviewees who observed the cows were asked if this has ever occurred again since the earthquake. Some people reported seeing the cows descending from the mountain at other times, such as when there was a sudden change in the weather, in particular the arrival of very bad weather or heavy snow. For this reason weather data were analyzed to rule out unusual weather as a cause of the cow movements.

### 3.2. Other Macroscopic Anomalies

A systematic literature search was carried out using scientific databases (ISI Web of Knowledge and Google Scholar), to find unusual behaviour of free ranging cattle. The search terms used were “cow” OR “cattle” AND “unusual behaviour/behaviour” which brought up more than 1000 documents, most of which were not relevant. Many of the observations of unusual behaviour of cattle prior to earthquakes related to restrained animals in domestic situations, and the general reaction was panic and attempts to escape. These were not included. Eight observations were found which are summarised in Table 1. We have also included in this table the 26 September 1997 Colfiorito earthquake of M_w_ = 6.1, which was preceded by a strong foreshock of M_w_ = 5.8.

**Table 1:** A summary of reports of unusual behaviour in free ranging cattle.

Behaviour	When behaviour occurred	Earthquake or possible explanation	Source
Cattle move to higher ground			
Cattle move to higher pastures	Few hours before earthquake and tsunami	27 March 1964, M = 8.7 Prince William Sound, Alaska. Offshore earthquake leading to tsunami flooding low lying meadows.	Engle, 1965 [28]
Cattle move to high ground	Before tsunami	26 December 2004. M = 9.1 Off shore earthquake off the coast of Aceh, Indonesia leading to flooding of low lying areas	Kelman *et al.* 2004 [29]
Cattle come down from hills			
Cattle come down from the hills	“shortly” before earthquake	18 April 1903. M = 8.3 San Fransisco, USA,	Lawson, 1908 [30]
Cattle come down from the hills	2 days prior to earthquake	26 September 1997. Colfiorito Earthquake of M = 5.8 (00:33 GMT) and M = 6.1 (09:40 GMT)	Fidani, 2013 [31]
Cattle leave high pastures on volcano	Just before volcano erupted	29 July 1968. Arenal Volcano erupted, Costa Rica	Anderson, 1973 [32]
Other unusual behaviour observed			
Cows enter suburb of major city	2 days prior to earthquake. Bukit Jalil, KL, Malaysia. April 9, 2012	11 April 2012. M = 8.6 Of the coast of Sumatra	Word Press, 2012 [33]
Hundreds of cows suddenly sit down in unison	Minutes before earthquake	22 February 2011. M = 6.3 Christchurch, New Zealand.	Whitehead and Ulusoy, 2013 [34]
28 cows die after falling over cliff	Lauterbrunnen, Switzerland	August 2009. No earthquake, thought to be related to violent thunderstorms	MailOnline, 2009 [35]
Cows exhibit no unusual behaviour			
Cows exhibit no unusual behaviour		16 December 2008. M = 4.7 Skåne, Sweden. Small earthquake, with different process to those expected to produce large scale ionisation	Haines, 2009 [36]

**Figure 3:**
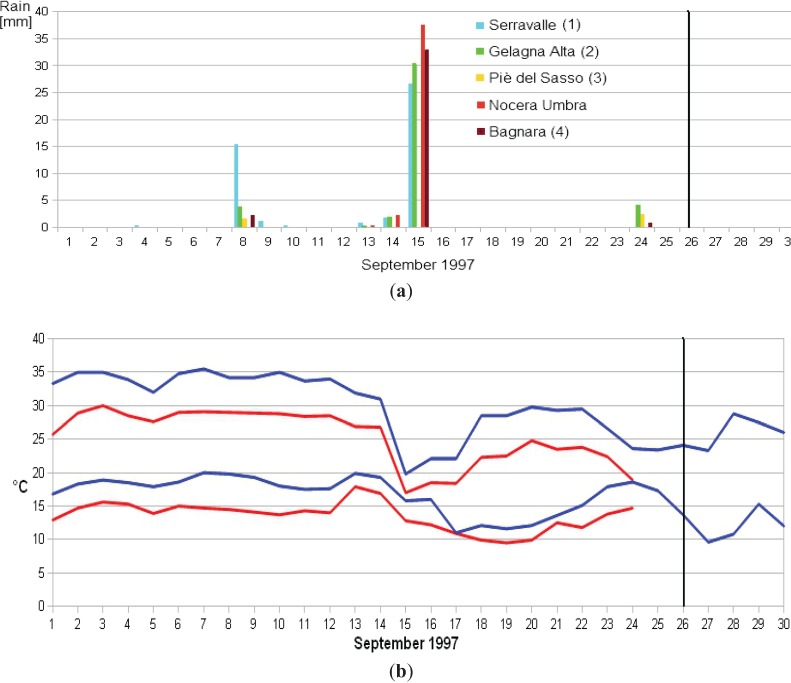
Rain data from the five meteorological stations. (**a**) rain data after September 22 were lost from Serravalle as it was strongly damaged by the quake; minimum and maximum daily temperatures recorded at Nocera Umbra, in red, and at Foligno, in blue, meteorological stations (**b**) temperature data after September 24 were lost from Nocera Umbra as it was strongly damaged by the quake; the main shock time is indicated by a black vertical line.

Meteorological data were retrieved from the Hydrogeological Annals of the Marche Region [37] and Umbria Region [38], from five stations around Colfiorito, (blue circles in Figure 1), and data were recorded with daily samples, (Figure 3a). Temperatures were reported in Figure 3b from the two stations closest to the epicentre. Gas emission data were retrieved from publications [39,40,41,42] and were also reported by the people filling in the questionnaires [31].

Earthquake lights (EQL) were also recorded in the vicinity. For example [31], light flashes were observed from Annifo above this village, few kilometers west of Monte Maggio, about two days before the main shock; and red sky was observed during the darkness from the Taverne village in the Colfiorito Plain, a few kilometers south of Monte Maggio, the night of the earthquake after the first shock at 2:33 LT (M_w_ = 5.8) and several hours before the main shock at 11:42 LT (M_w_ = 6.1). EQL are common phenomena for moderate earthquakes in Central Italy. The first collection of EQL data reported fire columns and red sky prior to many strong historical earthquakes [43] and contained the first classification based on shapes, colours, and time. Frederic Montandon proposed a revised classification, which reduced Galli’s number of EQL types from nine to five [44]. The first EQLs were photographically documented in 1966 along with a report containing numerous testimonials during an earthquake swarm near Matsushiro [45]. A review of observations has highlighted the well-established existence of luminous phenomena plus proposed theories [46]. A recent study on positive charge generation in igneous rocks opened the way to explaining luminous and other phenomena in a single coherent physical model [47]. Charge accumulations at asperities in the crust can produce corona discharges accompanied by the emission of light [48].

## 4. Theoretical Background

Rocks are generally good insulators. However, all rocks in the Earth’s deeper crust contain minerals, with point defects that had not been previously recognized. These defects are peroxy links such as O_3_Si/^OO^\SiO_3_ replacing the usual O_3_Si/^O^\SiO_3_ bonds. They are unusual because, in their normal state, they are dormant and electrically inactive. However, when rocks are stressed, peroxy links break and release mobile, positive electronic charge carriers. These are defect electrons in the O^2–^ sub-lattice, chemically equivalent to O^–^ in a matrix of O^2–^, known as positive holes and symbolized by h^•^ [49]. Positive holes are highly mobile. They are capable of flowing out of the stressed rock volume in which they were activated, spreading into the surrounding less stressed or unstressed rocks. They travel fast and far, tens of kilometers through the Earth’s crust, prior to large earthquakes maybe as much as hundreds of kilometers. As the h^•^ flow, they form electric currents in the Earth’s crust, often transient current pulses, but less frequently persistent fluctuation trains lasting hours or longer and tens to hundreds of thousands amperes strong [50]. When these h^•^ currents fluctuate, they emit electromagnetic (EM) radiation, of which the ultra low frequencies (ULF) and extremely low frequencies (ELF) can be transmitted through tens of kilometers of rocks and detected at the Earth’s surface. When the h^•^ arrive at the Earth’s surface, situations arise, which may be very relevant to the response of animals.

As their name suggests, positive holes are positive charges. Inside the Earth they repel each other electrostatically and “try” to get away from each other as far as they can. The Earth’s surface is as far as they can go, but even at the Earth’s surface the h^•^ will seek out topographic high points. They accumulate just below the surface, forming a positive charge layer with thickness d, generally some tens of nanometers. This subsurface charge layer is associated with a surface potential, V, typically on the order of 2–3 V.

As the rocks deep below around the hypocenter of the future earthquake, experience increasing levels of stress, ever more h^•^ charge carriers are activated. They will find their way to the surface and contribute to the build-up of the subsurface charge layer. The more h^•^ arrive, the more they compress the subsurface charge layer. Of special interest is the electric field E associated with such surface charge layers.

The electric field E is defined as potential V divided by the thickness d, E = V/d. If V reaches, say, 2 V and the thickness d of the h^•^ subsurface charge layer is, say, 100 nm (1 × 10^–6^ cm), the E field at the surface will be on the order of 2,000,000 V/cm. As the thickness d of the subsurface charge layer shrinks with the arrival of more h^•^, the E field grows. For instance, if V becomes 3 V and d shrinks to 30 nm (3 × 10^–7^ cm), the E field will be on the order of 10,000,000 V/cm. Under the effect of such steep E fields, even when they are microscopic and only act at very short range, air molecules become polarized in contact with the surface—so much so that they can lose an electron to the surface and become ionized. During this process, known as field-ionization, the air molecules, most likely O_2_, transfer an electron to an h^•^ in the surface, *i.e.*, to an O^–^, converting it to O^2–^:

O^–^|_surface_ + O_2_|_air_ => O^2–^|_surface_ + O_2_^+^|_air_ (1)

Equation (1) describes a process that causes the near-surface air to become laden with positive airborne ions such as O_2_^+^. All it takes is a sufficiently large number of h^•^ charge carriers, stress-activated deep below, coming to the Earth’s surface above the future earthquake hypocenter and form subsurface charge layers that generate the steepest E fields. Since the h^•^ repel each other electrostatically, they will seek out the higher elevations. Hence, if an earthquake is preparing to strike in a mountainous area, the reaction described by Equation (1) is most likely to start in the hills.

**Figure 4:**
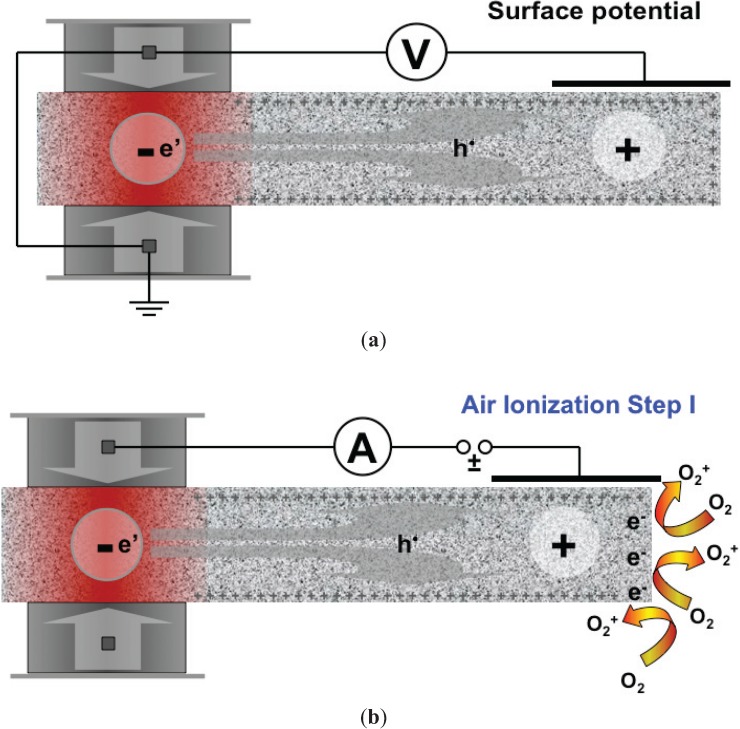
Laboratory set-up to measure surface potential (**a**) and air ionization (**b**) during stressing a block of rock [16].

Using an experimental set-up as depicted in Figure 4a it has been shown that, when a block of rock is mechanically stressed at one end, a positive surface potential develops at the other end that can be recorded by a voltmeter attached to a metal plate held 2 mm above the rock surface. Upon stressing the rock the surface potential rapidly increases to values around 2–3 V. Upon further loading of the end of the rock, the air in contact with the rock surface at the far end becomes positively ionized as indicated in Figure 4b [16]. This can be recorded by replacing the voltmeter in Figure 4a with an ammeter and insert a battery into the circuit so that the metal plate, held 2 mm above the rock surface, now acts as an ion collector. The number of positive airborne ions generated on the surface of a chunk of bare rock at moderate loads, typically less than 50% of the fracture strength, can reach values on the order of 10^6^–10^7^ cm^–2^·sec^–1^. Though we don’t know yet how these laboratory ionization production rates (in number of ions produced per unit surface) translate into actual air ion concentrations (number of ions per unit volume) in the field, data from Japan [51] indicate that positive air ion concentrations at ground level can be 2–3 orders of magnitude higher than the “fair weather” ion content, typically 200 cm^−3^, which is primarily due to cosmic rays and radon decay products [52]. Field data recorded by air ionization sensors collocated with the ULF search coil magnetometers of the QuakeFinder stations along the San Andreas Fault system in California and along the subduction zone in southern Peru have recorded massive pre-earthquake air ionization, often lasting for hours, producing predominantly or exclusively positive air ions [53,54].

It should also be noted that the generation of positive airborne ions is only Step I of the air ionization. When more h^•^ arrive due to the continuing build-up of tectonic stresses deep below, Stage II can set in which produces even more air ions, but now both positive and negative. The reason is that, with further increase in the number of h^•^ in the subsurface charge layer, the surface potential increases and the thickness of the surface/subsurface charge layer decreases. If the potential V reaches, say, 3 V, and the thickness d is reduced to, say, 6 nm, the E field would reach values on the order of 50,000,000 V/cm. Such high fields, even though they may act only over micrometer distances or less, can accelerate free electrons, which are always present in the ambient air due to ionization by cosmic rays and radon, to such high velocities that they begin to impact-ionize air molecules in their path. Such impact ionization events, which are likely to take place above sharp points, edges and corners, produce avalanches of electrons and positive ions, albeit in tiny air volumes, which trigger corona discharges.

**Figure 5:**
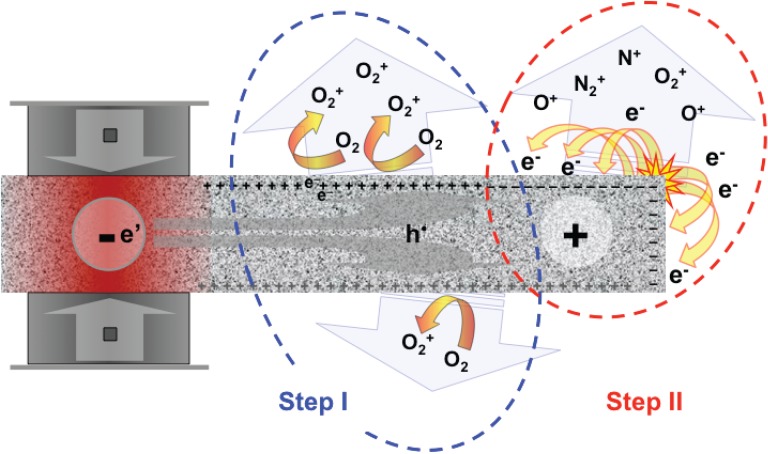
Schematic representation of the 2-step air ionization that has been demonstrated in laboratory experiments during stressing of rocks [16].

Indeed, tiny, rapid-fire 1.5 millisecond light flashes originating at the edges and corners of blocks of rock have been recorded in laboratory experiments as schematically shown in Figure 5 [16], generating bursts of free electrons and positive ions in roughly equal numbers. There seems to be no study cited in the literature that looked at the response to animals to an increase in the total air ion content, both positive and negative in equal proportion, compared to selectively positive air ionization. Animals might be sensitive to an overall increase in air ion concentrations but probably less so than to increases in selective positive air ionization. Furthermore, reaching the surface the charge cloud causes dielectric breakdown at the air-rock interface, *i.e.*, corona discharges, accompanied by the emission of light and high frequency electromagnetic radiation [16]. The light emissions observed at Colfiorito could have originated for the same physical process. Therefore this paper provides a small piece of evidence for Freund’s [55] unified theory of earthquake precursors. 

A parallel effect might have to be considered. As with any corona discharge in air, the flashes do emit light, which spans a broad spectrum over the VIS and UV regions, including “hard” UV. The hard UV portion will produce ozone, O_3_, and other reactive gases such as nitrous oxides, to which animals might also respond [56].

## 5. Discussion

It has been speculated that unusual animal movements could be caused by sound and ultrasound, such as before the Sumatra 2004 tsunami [57]. Sounds and explosions were also heard by the Colfiorito population, but these occurred months before the main shock [31] and no cow movements were observed at that time. The arrival of P waves could provide a possible causal link to many forms of unusual animal behaviour [58]. However P waves arrive only a few seconds before S waves, while the cows migrated down from the mountain two days before the quake, on September 24, 1997. The possibility of a P wave effect from a strong foreshock can also be ruled out, as the only foreshock (of magnitude 4) struck September 4, 1997, with no unusual cow movements in evidence. It is also highly unlikely that cows in Western Europe would move to avoid a predator. Cows are large animals and of course do have predators, but these are mainly large carnivores such as lions, panthers *etc.* that are not found in Central Italy. There are wolves and bears in some national parks of central Italy but they are few in number (having been reintroduced as a conservation effort) and they would not target a whole herd of adult cows.

Meteorological data indicate that the cattle movements were not caused by unusual weather. Figure 3a shows that abundant rainfall occurred on September 15 and slight rainfall on September 24. Temperatures reported in Figure 3b were within the norms of the season, which was verified by calculating weekly averages at the Foligno station where data had been available for 20 years. Maximum daily temperatures did not exceed two standard deviations, whereas minimum daily temperatures did not drop below two standard deviations. Both meteorological stations were not on the Colfiorito Plain, but in Nocera Umbra and Foligno at distances of 10 km and 20 km from the epicenter respectively. An intense and concentrated episode of evaporation reported on some occasions [1] and demonstrated in certain conditions [59], cannot be excluded. If, however, evaporation occurred it would not have been due to weather conditions, which usually affect regions much greater that the thermometric station distances from the epicentre considered here.

Positive airborne ions have long been known to have a strong physiological effect on animals and humans [60,61]. Therefore, it should not come as a surprise that animals on the ground and in the air, when exposed to high levels of positive airborne ions, would respond in ways which would minimize their exposure. In the case of the cows that left their pasture on top of Monte Maggio prior to the September 26 earthquake to seek refuge in Serravalle del Chienti, the unusual interruption of their summer grazing routine appears to be consistent with the known physics that controls the flow of stress-activated h^•^ charge carriers in an area of impending earthquake activity. Since the h^•^ charge carriers repel each other in the rocks as they spread toward the surface and “seek out” topographic heights, the air ionization process will begin with positive airborne ions generated massively on hilltops and other high elevation points.

The fact that the herd of cows left its high-lying summer grazing grounds to seek refuge in the valley is consistent with the expected elevation-dependent concentration gradient of positive airborne ions. As more and more stress-activated h^•^ charges arrive from below, the positive air ionization will probably spread to lower elevations, including the valleys. There is little recent research on the effects of positive ions on biological systems, however, research conducted in the 1960s and 1970s showed that positive air ions cause irritability and impaired motor abilities probably due to the fact that positive ions increase serotonin levels [61,62]. High levels of serotonin can also be caused by some antidepressant medication, so there has been much recent research on the dangers of high serotonin which can lead to serotonin syndrome, a potentially fatal condition. Symptoms of serotonin syndrome in humans include confusion, agitation, tremors or involuntary movements, akathisia (moderate to severe restlessness), hyperactivity, anxiety, and physiological symptoms such as hypertension and diarrhea, hypothermia [63,64,65].

Other pre-earthquake non-seismic phenomena may potentially have a noticeable effect on biological systems. In addition to the release of massive amounts of air ions, other gases have been reported to be released pre-seismically. One of the most toxic gases most likely to affect organisms is carbon monoxide [66]. Preseismic and coseismic geochemical variations were detected in some gas vents and natural springs during the 1997 seismic crisis in central Italy, interpreted as rate variations possibly due to crustal permeability changes [39]. Dissolved gases at nearby springs exhibited a slight enrichment of dissolved CO_2_ and CH_4_ after the main shock [40]. It has been suggested that increased CO_2_ pressure triggered aftershock activity by significantly reducing the effective normal stress [41].

CO data were collected only after September 1997 in Central Italy [42] and there are no data relative to the period of August–September 1997.

Carbon monoxide is highly toxic because it deprives the body of vital oxygen. Carbon monoxide binds preferentially with haemoglobin (the substance which transports oxygen in the blood stream) and prevents the O_2_ being carried to the tissues. Carbon monoxide’s affinity for haemoglobin is 200 times that of oxygen, meaning that it will bind preferentially even at relatively low concentrations, leading to the formation of carboxyhaemoglobin and shifting the oxyhaemoglobin dissociation curve leftwards. As a result of follow-on reactions in the organism the result is hypoxia which can lead to death of the organism [67,68]. Symptoms of CO poisoning in humans include headache, nausea, and dizziness, confusion, weakness, and in more severe cases, respiratory and cardiac failure and eventually, coma [69].

According to the questionnaire, sulfurous gas emissions in the Colfiorito area seem to have been concentrated in the valley floor, thus they are unlikely to have been the cause of the unusual cow movements down the mountain.

Although avoidance tests have not yet been specifically carried out with positive air ions, carbon monoxide and sulfurous gases, animals will generally seek to avoid and move away from harmful and toxic substances in their environment. For example, animals will generally show avoidance responses to pollutants in their environments [70], so much so that many organisms’ avoidance responses are used as bioassays for the presence of pollutants [71,72,73].

### 5.1. Behaviour of Domestic Cows and other Animals before Earthquakes

The cow (*Bos primigenius*) is a domesticated species of ungulate with a long association with humans for the purpose of providing meat and milk [74]. Cows are a species of animal that tends to follow a predictable routine, not generally deviating from it [74]. Cows have been reported for centuries to exhibit strange behaviour prior to earthquakes [1]. Twenty to thirty hours before the Lisbon earthquake, November 1, 1755, cattle became very excited. Excessive excitement, panic or vocalization of cattle has been observed before many other earthquakes as well including that in Naples, Italy in 1805; Fruili, Italy on May 6, 1976 and the 1907 Karatagh earthquake in the border area between Uzbekistan and Tajikistan. Cattle have been reported to refuse to enter their stalls (Tangshan earthquake of May 25, 1970), to behave aggressively and to attack each other (Haicheng earthquake of February 4, 1975) [1].

Of interest are the two different types of behaviour exhibited by cows (aggressive or excited, as opposed to simply moving between high and low areas). Domestic animals are often reported to panic and break free of tethers, or to become aggressive, agitated or restless, whereas wild animals are usually reported to leave an area and move somewhere else. In the book on animal behaviour and earthquakes by Tributsch [1], wild animals are reported to show unusual movements, whereas domestic animals mainly show restlessness, aggression and attempts to escape (summarised in Table 2). This could be interpreted as animals moving away from aversive stimuli to other areas, and those prevented from doing so start to panic or attempt to escape. This observation, along with others of cow movements prior to natural disasters, supports the hypothesis [75] that unusual behaviour prior to earthquake is primarily a movement away from toxic or aversive stimuli. As discussed, in terrestrial animals, these are likely to be gasses such as CO released from the fault [3] or high concentrations of positive air ions [16].

**Table 2:** Summary of Reponses of wild *vs.* domestic/captive animals prior to earthquakes (adapted from Tributsch [1]).

Wild or Unrestrained Animals	Domestic or Captive animals
**Birds** • behave fearfully or agitated • take to the air en masse • leave the trees, nests or dovecote • vocalising excessively • leave usual habitat • giant flocks seen	**Dogs** • barking, whining or howling excessively • fearfully, agitated or restless • escaped/lost
**Rats and/or weasels ** • fleeing the town or city • appear in packs • leave houses and granaries or other buildings • run around town	**Horses ** • behaving fearfully, agitated or restless • stamping, kicking or rolling on the ground. • impossible to ride or refusing to walk • breaking out of halter or tether • vocalising excessively • escaped/lost
**Reptiles and amphibians** • Snakes leave their burrows in winter/crawl on snowy ground • Snakes seen in large numbers • Turtles jump out of water • Lizards come out of their burrows • Amphibians leave their breeding site	**Domestic birds (Hens/Chickens/Geese)** • refuse to enter coops • behave fearfully or agitated • excessively noisy • Cockerels crow all night long • break out of enclosure or try to escape • escape/lost
**Invertebrates ** • Swarm of bees leave the area • Ants leave holes • Sea cucumbers disappear • Large numbers of earthworms leave the soil • Large numbers of sea urchins appear • Large crab migrations/crabs crawl to shore • Plankton comes to the surface • Large number of flying ants seen • Swarms of millipedes are seen • Sea snakes swim upriver • Lobster and squid caught at surface • Many octopuses in shallow water or acting strangely	**Cattle/pigs/sheep ** • behave fearfully or agitated, restless • refuse to enter stalls • vocalising excessively • become aggressive, bite each other • try to break out of enclosures • escape from enclosures/lost **Zoo animals ** • behave fearfully, excited or restless • refuse to enter pens or enclosures • try to breakout of enclosures • escape/lost

The literature search and the observation at Colfiorito show that the behaviour of free ranging cows before natural disasters is highly context specific. Cows have been shown to adjust their movements before earthquakes, tsunamis, volcanoes and other natural disasters. Kirschvink [58] discusses the possibility of animals having an evolved seismic escape response. This is plausible if movement to higher or lower ground before natural disasters increased survival chances, leading to a genetic predisposition to avoid stimuli which are reliably linked to future natural hazards. By way of a Monte-Carlo simulation, Kirschvink [58] shows that rare events such as earthquakes can drive evolutionary change if the consequences are severe enough. However, it is more likely that the more parsimonious explanation is true; animals which behave unusually or adjust their positions before earthquakes are simply moving away from aversive stimuli in their environment. Cows responding to volcanic eruptions and tsunamis may be responding to infrasound, although no evidence exists at present for the stimuli that animals may respond to prior to these particular hazards. 

The cause of the stimuli that the animals are avoiding may be different for different earthquakes. For example, Whitehead and Ulusoy [34] suggest that the cows, which changed their body position prior to the Christchurch earthquake, were acting to ease discomfort brought on by exposure to ULF radiation. Ikeya 2004 [76] reports similar behaviour prior to the Gujarat Earthquake in elephants in the nearby Ahmedabad Zoo. Pre-seismic ULF emissions and their likely effects on biological systems provide an additional possible mechanism for the unusual cow movements [9]. Cows are known to align their bodies along the N-S geomagnetic axis. This effect can be disrupted by overhead power lines and their associated magnetic fields [77]. Hence, a possible explanation for unusual cow movements at Colfiorito could be due to ULF magnetic field anomalies, which have been reported to occur before large earthquakes [58]. However, for the Colfiorito earthquake, no convincing evidence for ULF magnetic anomalies in the range of 4–100 mHz was recorded by the geomagnetic station at L’Aquila [78], which is about 80 km from the epicentre of the main shock, making the positive holes hypothesis much more plausible for this particular earthquake.

There is a report of cattle showing no recognizable unusual behaviour before a small earthquake in Sweden on December 16, 2008 [79]. However, this event occurred at 20 km depth and had a magnitude of only M = 4.7, confirming that unusual animal behavior is generally not observed prior to smaller earthquakes M < 5. Furthermore, not every earthquake causes unusual animal behaviour [80], which may be due to differences in geology, focal mechanisms and the specific behaviour of individual animals.

### 5.2. Possible Cause of Cows Leaving Their Pastures Prior the Colfiorito Earthquake

**Figure 6:**
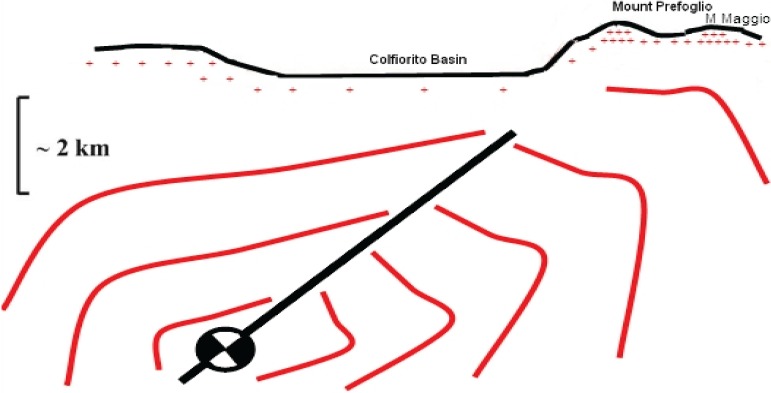
Surface profile extracted from Figure 1b, showing the Colfiorito plain, Monte Prefoglio (and Monte Maggio behind it) relative to the fault geometry which generated the Umbria-Marche earthquake; + symbol represents the charge distribution on the ground surface.

We have shown that unusual movements of free ranging cattle are not uncommon before earthquakes and other natural hazards (Table 1). The particular case of the Umbria-Marche earthquake, where the tectonic structure of Colfiorito area is well known, suggests the importance of the relative position of Monte Maggio to the fault geometry and position (Figure 6). Even leaving aside the water-saturated sediments in the valleys and the Colfiorito basin, which may inhibit transmission of the electronic charge carriers by ionizing water molecules at the water-rock interface, the density of airborne positive ions will increases on mountain tops. Charge density on the ground is represented by the + symbol density on Figure 6. The earthquake focal mechanism of the Colfiorito fault shows a region of compressive stresses towards Monte Maggio, probably intensifying the flow of charges in that direction. The red lines in Figure 6 represent estimated fair potential lines.

## 6. Conclusions

Cow movements two days before the Colfiorito, September 26, 1997 Central Italy earthquake, were observed by numerous people in the village of Serravalle del Chienti. As the recorded rainfall and temperature in September 1997 were within seasonal averages, it is unlikely that the cow movements were due to these factors. 

The movement of unrestrained animals (such as the cows at Colfiorito) away from their usual habitat which has been observed frequently [1,81] and the excited and agitated way in which they reportedly behave when prevented from leaving an area, may be due to frustration on attempting to escape from the aversive environmental conditions or could be due to the serotonin syndrome. This could be caused by an increase in positive airborne ions due to the arrival, at the ground-to-air interface, of charge carriers, activated deep below the Earth’s crust and spread over a wide area surrounding the forthcoming epicentre. The same charge excess could account for luminous effects and earthquake lights, which were also observed in the area.
